# Dentistry among the Speechless

**Published:** 1894-12

**Authors:** Charlotte E. Benton


### THE DENTAL REGISTER.

VOL. XLVIII.] DECEMBER, 1894. [No. 12.

### COMMUNICATIONS.

### Dentistry Among the Speechless.

BY CHARLOTTE E. BENTON, D.D.S.

In the part of New York City called Washington Heights, overlooking the Hudson, is the group of large buildings known as the "New York Institution for the Instruction of the Deaf and Dumb." Ample provision is made for the teaching, health and general well-being of the pupils, who number about four hundred.

A year and a half ago a dentist was added to the staff. This innovation was for a time looked upon by the pupils with little favor, but after each pupil had been summoned to the dreaded chair and every obnoxious root removed, the dismay subsided somewhat. As the filling progressed and the consequent relief from pain was experienced, the silver lining of the dubious cloud became apparent and the path of the deaf mute did not seem so dark, even when it led to the dental chair. One of the buildings is called the "Mansion House," I suppose because it was many years ago the country-seat of a nephew of President Monroe. Its stately rooms are now the home of over eighty little boys all under twelve years of age. Their reception of the dental idea was amusing. Each, as he came from the hands of the dentist, turned loose once more among his fellows, was the center of an eager and somewhat anxious crowd. Twenty pairs of eyes looked intently at his open mouth, seeing each filling and noting especially any extractions which had been made, and their fingers flew too fast for any but a practiced eye to follow, asking all about how it was done. So well did they learn that more than once I was startled to see a child, who had never been in a dentist's chair before, tell me in signs that he preferred gold to gutta-percha, and suggesting not to use the engine.

*I love to fill children's teeth*, and I find them very good patients. The extraction was very conservative and every tooth that could be was made serviceable. This made about two hundred root-canals to fill during the first three months. During the fifteen months which have passed since, five of these teeth have given trouble. Two of them I was unable to subdue and they were extracted. Two others, upper molars, caused slight swelling and pain though not sufficient to keep the patient awake at night. A small fistula appeared from a buccal root and the symptoms disappeared, the whole trouble having arisen and subsided in forty-eight hours. The fifth was comfortable for six months when it gave pain. Removing the filling and a further portion of the crown of the tooth, one root was found not well filled. The canals were cleaned and refilled and the tooth has been all right for a year past. The canals are always kept dressed for a few days with an antiseptic adapted to the case, after the nerve is removed, before filling.

I have great faith in hot water and hot air as antiseptic and purifying agents. The root canals have been filled with gutta-percha, in one form or another, after drying them thoroughly with heated broaches.

The various diseases which have deprived the mute of hearing, have also lessened the sensitiveness to pain, and thirty exposed pulps were capped, hoping the effort might be more successful than with hearing and speaking people. Of these capped pulps nine have died. Probably the anæmic condition of many of the patients is one element against success. The capping was done at the same time as the root-canal filling. Quite a proportion of them had what are termed syphilitic teeth, or show the disease in the shape of brow and eyes. Adenoid growths and enlarged tonsils are common.

Children five years of age are admitted to the Mansion House for kindergarten training. When ten or twelve they go into the regular course of study which is eight years long. This gives a good opportunity of testing different methods with a chance to observe the results at any time. Nearly all cavities are lined with a non-conductor, usually gutta-percha, before filling, otherwise the pupils return complaining of thermal shock. Over two thousands fillings have been put in for them. But very few of these have failed as yet, and not one of these failures can I attribute to the presence of the gutta percha.

Of cases which I do not understand I will mention these. A little girl erupted the lower bicuspids of the left side of normal appearance, but the first began to loosen before it had been in place six months. Then a brown spot appeared on the distal side of the crown, and the gum was inflamed by the irritation of the loose tooth. Last June the child went home. Returning to school this Fall, I found the tooth gone, and the process also—the space like a jaw from which the teeth have been gone a long time. Replying to my questions, she signed to me that it did not hurt, but was "weak" and a man pulled it out. The deciduous molars of the right side are still in place. The remaining bicuspid is not affected.

A young man came up saying he wished to see me the day before, as he was suffering very much, but I was not there. The lower jaw was badly swollen on one side opposite the molars. He said it was the gum which pained, not the teeth. Examination showed he was correct. The mouth was packed with cotton and the swollen portion wiped dry. It was hard but not red. Pressure caused an exudate of a turbid, yellow fluid, resembling bile, coming from the substance of the gum, not from the tooth socket.

I did not treat it. In three days the symptoms disappeared. The patient has good teeth and good health. Another young man with large, strong teeth, has the upper molars exposed almost to the bifurcation of the roots. I cannot discover any pus or unhealthy state of the gums, and the teeth are not loose.

The front and all the lower teeth are normal.

